# Efficacy of metabolites of a *Streptomyces* strain (AS1) to control growth and mycotoxin production by *Penicillium verrucosum*, *Fusarium verticillioides* and *Aspergillus fumigatus* in culture

**DOI:** 10.1007/s12550-020-00388-7

**Published:** 2020-01-20

**Authors:** A. Mohd Danial, A. Medina, M. Sulyok, N. Magan

**Affiliations:** 1 Applied Mycology Group, Environment and AgriFood Theme, Cranfield University, Cranfield, Bedford, MK43 0AL UK; 2 Present Address: Science and Food Technology Research Centre, Malaysian Agricultural and Research Institute, 43400 Serdang, Selangor Malaysia; 3 Institute of Bioanalytics and Agro-Metabolomics, Department of Agrobiotechnology (IFA-Tulln), University of Natural Resources and Life Sciences, Vienna, Konrad Lorenzstr. 20, A-3430 Tulln, Austria

**Keywords:** *Streptomycetaceae*, Antifungal metabolites, Fungal pathogens, In vitro efficacy, Mycotoxins, Germination, Mycelial growth

## Abstract

The objectives of this study were to determine the efficacy of metabolites of a *Streptomyces* strain AS1 on (a) spore germination, (b) mycelial growth, (c) control of mycotoxins produced by *Penicillium verrucosum* (ochratoxin A, OTA), *Fusarium verticillioides* (fumonisins, FUMs) and *Aspergillus fumigatus* (gliotoxin) and (d) identify the predominant metabolites involved in control. Initial screening showed that the *Streptomyces* AS1 strain was able to inhibit the mycelial growth of the three species at a distance, due to the release of secondary metabolites. A macroscopic screening system showed that the overall Index of Dominance against all three toxigenic fungi was inhibition at a distance. Subsequent studies showed that the metabolite mixture from the *Streptomyces* AS1 strain was very effective at inhibiting conidial germination of *P. verrucosum*, but less so against conidia of *A. fumigatus* and *F. verticillioides*. The efficacy was confirmed in studies on a conducive semi-solid YES medium in BioScreen C assays. Using the BioScreen C and the criteria of Time to Detection (TTD) at an OD = 0.1 showed good efficacy against *P. verrucosum* when treated with the *Streptomyces* AS1 extract at 0.95 and 0.99 water activity (*a*_w_) when compared to the other two species tested, indicating good efficacy. The effective dose for 50% control of growth (ED_50_) at 0.95 and 0.99 *a*_w_ were approx. 0.005 ng/ml and 0.15 μg/ml, respectively, with the minimum inhibitory concentration (MIC) at both *a*_w_ levels requiring > 40 μg/ml. In addition, OTA production was completely inhibited by 2.5 μg/ml AS1 extract at both *a*_w_ levels in the in vitro assays. Ten metabolites were identified with four of these being predominant in concentrations > 2 μg/g dry weight biomass. These were identified as valinomycin, cyclo(L-Pro-L-Tyr), cyclo(L-Pro-L-Val) and brevianamide F.

## Introduction

There has been interest in the utilization of actively growing natural microorganisms for the competitive exclusion of toxigenic fungal species or by using their naturally produced metabolites for inhibiting the germination and growth of these pathogens that cause diseases of humans and contaminate food and feed (Dogi et al. 2013; Faheem et al. 2015; Guo et al. 2011). A key driver is the strict legislative limits, which exist in many countries for mycotoxins in a range of raw and processed staple commodities. Thus, minimization strategies are actively being sought to reduce potential exposure to spoilage and toxigenic moulds in food and feed chains. There is thus interest in the identification of natural compounds that may control or reduce mycotoxigenic mould colonization and toxin contamination of staple commodities.

Food and feeds such as cereals, nuts and spices are commonly contaminated with mycotoxigenic fungi and mycotoxins that are a serious problem faced by many countries (Lee and Ryu 2017). This causes significant losses to producer countries when their exports are rejected because they do not meet the legislative limits, especially in Europe. The Rapid Alert System for Food and Feed shows that up to 30% of commodities imported into the EU are rejected because of mycotoxin contamination (RASFF 2018). Species from the genera *Penicillium*, *Fusarium* and *Aspergillus* are of greatest concern in terms of mycotoxin contamination of food and feed. A survey conducted in 2014 revealed that more than half of the worlds’ regions were severely affected by mycotoxins including fumonisins (FUMs), deoxynivalenol (DON) and zearalenone (ZON) from *Fusarium* species which had increased when compared to 2013 (Kovalsky 2015). Moreover, the maximum and average ochratoxin A (OTA) concentration in samples from Europe in 2017 was the highest when compared to other regions (Biomin 2018). In a 4-year survey by Limay-Rios et al. (2017) of stored wheat in Canada, *Penicillium verrucosum* was commonly isolated, as well as contamination with OTA, ochratoxin B (OTB) and citrinin.

*Aspergillus fumigatus* is an opportunistic fungal species, responsible for aspergillosis due to lung infection. Resistance to azoles has resulted in a number of virulent strains which have become difficult to control (Chowdhary et al. 2013), especially in immunocompromised age groups (Steinbach et al. 2012). *A. fumigatus* also produces the mycotoxin gliotoxin (GLI) (Kupfahl et al. 2008). Indeed, in Manchester, UK, azole-resistant strains were first detected in 1999 (Howard et al. 2009) and are now commonly found in Switzerland, USA, India and China (Hurst et al. 2017; Lockhart et al. 2011; Riat et al. 2018). However, at the present time, no regulations exist with regard to GLI exposure.

*Streptomyces* species are gram-positive filamentous bacteria, which can grow in various ecosystems, including sea sponges (Han et al. 2009), soil (Nguyen et al. 2015), animal faeces (Wang et al. 2014) and termites (Zhang et al. 2013). They are able to produce both secondary metabolites (Wang et al. 2013; Zhang et al. 2013) and hydrolytic enzymes (Karthik et al. 2015; Nagpure and Gupta 2013) or potentially novel anti-microbial compounds (Yang et al. 2015; Yekkour et al. 2015; Shakeel et al. 2018). Some compounds have been shown to inhibit spore germination (Wang et al. 2013; Zhang et al. 2013) and mycelial growth of spoilage fungi (Nguyen et al. 2015). However, many studies have screened metabolites for efficacy only against mycelial growth with less emphasis on control of mycotoxin production or in relation to different interacting environmental conditions. There have thus been significant research efforts to screen, isolate and identify novel compounds with antifungal activities from *Streptomyces* strains.

Previously, Sultan and Magan (2011) examined a *Streptomyces* strain (AS1) isolated from peanuts. This was found to be competitive and some extracts from the culture were found to be very effective at inhibiting *Aspergillus flavus* and aflatoxin B_1_ production, both in vitro and in stored peanuts. However, this *Streptomyces* strain and its metabolites have not previously been screened against other spoilage toxigenic moulds or indeed against any human fungal disease-causing pathogens or for control of toxin biosynthesis. In addition, the compounds responsible for the inhibition were not previously identified. The objectives of this study were to determine the efficacy of the *Streptomyces* AS1 metabolites on (a) spore germination and mycelial growth of *P. verrucosum*, *Fusarium verticillioides* and *A. fumigatus*, (b) efficacy for control of the production of OTA, FUMs and GLI and (c) to identify the major metabolites produced by the *Streptomyces* AS1 strain responsible for the control achieved.

## Materials and methods

### Bacterial strain

A *Streptomyces* AS1 strain was obtained from the Applied Mycology Collection, Cranfield University. This strain was previously isolated from Egyptian peanuts by Dr. Y. Sultan (Sultan and Magan 2011). This strain was subsequently identified as *Streptomyces parvus* based on molecular analyses (99%; EU accession number: EU841619.1).

### Mycotoxigenic fungal strains

*Fusarium verticillioides* was from the Applied Mycology Collection, Cranfield University. It was isolated from maize by Dr. N.I.P. Samsudin and molecularly identified (Samsudin et al. 2017) and *Penicillium verrucosum* (OTA11) and *Aspergillus fumigatus* (strain Mi538) were kindly provided by Dr. M. Olsen (National Food Administration, Uppsala, Sweden). These fungal strains have all previously been demonstrated to produce high titres of their respective toxins (Cairns-Fuller et al. 2005; Samsudin et al. 2017).

### Preparation of spore suspensions

#### *Streptomyces* AS1 and test mycotoxigenic fungi

A glycerol stock solution of *Streptomyces* AS1 was inoculated on half nutrient agar (½NA) and incubated at 25 °C for 5 days or until sporulation had occurred. The colonies were flooded with sterile 10 ml of 0.1% (w/v) Tween-80/water solution and harvested by gently scraping the colony with a sterile spreader to release the spores and this was then transferred aseptically into a sterile 50-ml conical tube and the density adjusted to 1.0 at OD_600_ or approximate 10^8^ spores/ml.

*A. fumigatus* and *F. verticillioides* were grown on malt extract agar (MEA; Oxoid Ltd) and *P. verrucosum* on potato dextrose agar (PDA, Oxoid Ltd) for 7 to 10 days or until sporulation occurred at 25 and 30 °C (*A. fumigatus* only). Fungal spores were harvested by pouring 10 ml of sterile 0.1% Tween-80/water solution onto the agar surface containing the cells or spores and gently scraping with a surface-sterilized glass rod. The cell/spore suspensions were transferred into sterile 50-ml tubes, centrifuge at 2000 rpm for 2 min and the supernatants discarded. Fresh sterile 0.1% Tween-80/water was added. The concentration of fungal spores was counted using a haemocytometer (Thoma, Germany) and adjusted to approx. 10^6^/ml with sterile 0.1% Tween-80/water solution.

#### Spore germination assays: media preparation, inoculation and incubation

Molten cooled autoclaved ½NA was poured into Petri plates (90 mm ∅) in a sterile flow bench and allowed to solidify. The cooled media were overlaid with a sheet of sterile cellophane (8.5 cm diameter) carefully to avoid any air bubbles. A single colony of *Streptomyces* AS1 was streak plated on the ½NA previously overlaid with a sterile cellophane sheet and incubated at 25 °C for 10 days. At different time intervals (days 2, 5 and 10), the cellophane layer with the *Streptomyces* AS1 biomass was carefully removed and 100 μl of spore suspension (10^6^ spores/ml) of the test pathogens was spread plated onto the agar surface with a sterile glass spreader. These Petri plates were then incubated for 48 h. *P. verrucosum* and *F. verticillioides* were incubated at 25 °C and *A. fumigatus* treatments at 30 °C. After 24 and 48 h, two agar plugs (18 mm ∅) were taken randomly and placed on a glass slide. They were stained with lactophenol cotton blue, covered with a coverslip prior to microscopic examination. A total of 50 spores in each of four fields were counted in each replicate and the number recorded. Spores were considered germinated when the germ tube length was equal to or greater than the spore diameter (Magan 1988). The experiments were all carried out with three replicates per treatment and repeated once.

### Mycelial growth and mycotoxin assays

#### Preparation of cell-free supernatant for growth and mycotoxin inhibition assays

The *Streptomyces* AS1 spore suspension (200 μl) was inoculated into 200 ml ½ strength sterile nutrient broth (NB) and incubated at 30 °C at 200 rpm for 4 days. After 4 days, the supernatant was separated from the mycelium by filtration using Whatman filter paper no. 4.

#### Extraction of *Streptomyces* AS1 bioactive metabolites using ethyl acetate

Bioactive metabolites from the *Streptomyces* AS1 supernatant were extracted three times with ethyl acetate (EA) (Sultan and Magan 2011). Approx. 900 ml of cell-free supernatant was mixed with 300 ml of EA in a separating funnel and after shaking for a few seconds, the mixture was left to separate into two layers. The EA layer (upper layer) containing the bioactive metabolites was collected and the extraction phase was repeated three times. The EA layers containing the bioactive metabolites were combined and the solvent was removed using a rotary evaporator at 38 °C. The dried film was dissolved in DMSO under sterile conditions for performing the assays.

### Efficacy of *Streptomyces* AS1 EA extract against fungal pathogens using the BioScreen C turbidimetric assay

#### Preparation of culture medium

The culture medium used to inoculate the fungal pathogen was prepared as described by Medina et al. (2012). Water was used to prepare the medium and this was adjusted with glycerol to 0.99 and 0.95 water activity (*a*_w_). The culture media used were semi-solid YES (yeast extract sucrose) for *P. verrucosum* and *A. fumigatus* and PD for *F. verticillioides*. Each culture medium contained the following: (1) YES: yeast extract 20 g/l, sucrose 150 g/l, MgSO_4_.H_2_O 0.5 g/l and 0.05% agar (w/v) and (2) PD: potato extract 4 g/l, dextrose 20 g/l and 0.03% agar. Both culture media were sterilized at 121 °C for 15 min.

#### Culture medium containing AS1 ethyl acetate extract and spore suspensions

A mixture of 9800 μl culture medium, 100 μl of spore suspensions (final spore count 10^5^ spores/ml) and 100 μl of EA extract (final concentrations of 5 μg/ml, 10 μg/ml, 20 μg/ml, 30 μg/ml and 40 μg/ml; w/v) was prepared in sterile 25-ml universal bottles. A total of 300 μl of the mixture were loaded into 100-well microtitre plates and the density measured automatically at 600 nm every 30 min at 25 °C for 7 days for *P. verrucosum* and *F. verticillioides* and at 37 °C for *A. fumigatus*. This method was based on that developed for screening compounds for efficacy against filamentous fungi using the BioScreen C bioassay (Medina et al. 2012). Each set of experiments was carried out with ten replicates. The relative time to reach an absorbance value of 0.1 (time to detection; TTD-0.1) was then compared as an indication of growth rates. The raw data was analysed using Microsoft Excel to obtain the growth curve for the fungal species. The TTD of 0.1, minimum inhibitory concentrations (MIC) and IC_50_ concentrations were calculated using the Lambert-Pearson model (Lambert and Pearson 2000).

### Agar medium containing EA extract and inoculation of fungal spores

Approximately 100 μl of the different concentrations of the EA extract (0–40 μg/ml) was added aseptically into 10 ml sterile YES (for *P. verrucosum* and *A. fumigatus*) and PD (for *F. verticillioides*) agar (± 52 °C) with different *a*_w_ levels (0.99 and 0.95) and inverted several times before pouring into Petri plates (53 mm ∅). After the agar media had solidified, 100 μl of spore suspension (10^7^ spores/ml) was spread plated using a surface-sterilized glass spreader and incubated at 25 °C for *P. verrucosum* and *F. verticillioides* and 37 °C for *A. fumigatus* for 7 days. After 7 days, the agar plugs were collected for mycotoxin analysis.

### Extraction and quantification of ochratoxin A, gliotoxin and fumonisins

#### Ochratoxin A

A total of five agar plugs of *P. verrucosum* after 7 days growth on YES medium were randomly collected across the colony using a sterile cork borer (number 5) and transferred into pre-weighed 2-ml tubes and re-weighed to obtain the weight of the agar plug. The extraction of ochratoxin A (OTA) from the agar plug was carried out using 1 ml of methanol. The samples were shaken at 200 rpm and 30 °C in the dark for 1 h. Samples were then centrifuged at 15,000*g* for 5 min and the methanol containing OTA was filtered (nylon syringe filter, 0.22 μm pore size, Fisher) into amber and silanized HPLC vials (Agilent, UK) for analysis.

The separation and quantification of OTA was done using an Agilent 1200 Series HPLC system (Agilent, UK) with a fluorescence detector. The separation was done at 25 °C using a Poroshell 120 EC-C18 (4.6 mm × 100 mm, 2.7 μm) column fitted with a guard column (4 mm × 3 mm cartridge, Phenomenex, USA). The mobile phase was water:acetonitrile:acetic acid (41:57:2, v/v/v). The flow rate and injection volume were 1 ml/min and 20 μl, respectively. The detection wavelength was 333 nm for excitation and 460 nm for emission. Different concentrations of OTA standards (0–400 ng/ml) were prepared by dissolving OTA standard (Sigma) solution in methanol (*R*^2^ = 0.9994). The LOD and LOQ were 12.3 ng/ml and 41.1 ng/ml, respectively.

#### Gliotoxin

The agar plugs of the growing colonies were obtained as described previously for OTA. The extraction was carried out using chloroform (1 ml) for 1 h at 30 °C and shaken at 200 rpm. After this, 800 μl chloroform containing gliotoxin (GLI) was transferred into a new 2-ml tube and dried overnight in a fume cupboard. The dried extract was dissolved in 700 μl of mobile phase solution (1% acetic acid:acetonitrile (75:25, v/v) and filtered into amber and silanized HPLC vials for analysis.

The separation and quantification of GLI was done according to Alonso et al. (2016) with slight modification. The GLI concentration was measured using a reversed-phase HPLC system linked to a diode array detector (DAD). The separation was done at 25 °C using a Zorbax Eclipse XDB-C18 (4.6 mm × 150 mm, 5.0 μm, Agilent, USA) column fitted with a guard column (4 mm × 3 mm cartridge, Phenomenex, USA). The mobile phase was acetic acid:water (1:99, v/v) (eluent A) and acetonitrile (eluent B). The flow rate, injection volume and detection wavelength were 1.3 ml/min, 50 μl and 268 nm respectively. The gradient programme was 25% B for 10 min followed by rapid increased to 100% in 1 min and this was held for 8 min before decreasing this to 25% in 2 min. Working GLI standard solution with a concentration range from 0 to 3000 ng/ml was prepared by diluting GLI standard (Sigma) in mobile phase (*R*^2^ = 0.9999). The LOD and LOQ were 41 ng/ml and 138 ng/ml, respectively.

#### Fumonisins

Fumonisins B_1_ and B_2_ (FB_1_, FB_2_) are the main mycotoxins in the suite produced by *F. verticillioides*. The agar plugs were extracted by adding 1 ml of acetonitrile:water (50:50, v/v) and shaking the mixture at 200 rpm for 1 h at 30 °C. Acetonitrile:water (50:50, v/v) containing FB_1_ and FB_2_ was then filtered into amber and silanized HPLC vials for analysis.

The separation and quantification of FBs was done using the HPLC-FLD system (Agilent, UK). The separation was done using a Zorbax Eclipse Plus C18 (4.6 mm × 150 mm, 3.5 μm) column + a guard column (security guard, 4 mm × 3 mm cartridge, Phenomenex, USA) at 30 °C. The detection was at 335 nm for the excitation and 440 nm for the emission wavelength. The mobile phase was 50 mM NaH_2_PO_4_ (pH 4.01):methanol (50:50, v/v) (eluent A) and acetonitrile:water (80:20) (eluent B). The injection volume was 15 μl with a flow rate of 1 ml/min. The gradient programme was 0% B for 5 min, increasing B to 50% in 1 min and held for 7.10 min before slowly increasing to 80% in 6.90 min. Before injecting the standards or samples, 10 μl of standards or samples was mixed with 5 μl of derivatization solution (OPA). The derivatization solution consisted of 1 ml ortho-phthaldialdehyde (40 mg of OPA in 1 ml absolute methanol), 5 ml of 0.1 M Na_2_B_4_O_7_.10H_2_O and 50 μl of 2-mercaptoethanol. The derivatization was carried out with an auto-derivatization programme in the HPLC system. Different concentrations of FB standards (0–5 μg/ml) were prepared by diluting FB standard solution (Sigma-Aldrich, USA) in acetonitrile:water (50:50, v/v) (*R*^2^ = 0.9972 for FB_1_ and 0.9959 for FB_2_). The LOD and LOQ were 0.37 μg/ml and 1.21 μg/ml for FB_1_ and 0.44 μg/ml and 1.48 μg/ml for FB_2_, respectively.

### Identification of bioactive compounds from *Streptomyces* AS1 EA extract

Sample preparation, detection and quantification were performed as described by Malachová et al. (2014). Briefly, the extraction solvent (acetonitrile/water/acetic acid; 79/20/1) was added to dried ethyl acetate extract and after shaking and centrifugation, the extract was injected into LC-MS/MS equipped with a TurboV electrospray ionization (ESI) source. The Phenomenex C18-column (150 × 4.6 mm, 5 μm) fitted with a C18 security guard cartridge (4 × 3 mm) was used to separate the compounds. The mobile phases consisted of methanol/water/acetic acid with the ratio of 10/89/1 (v/v/v) for eluent A and 92/2/1 (v/v/v) for eluent B. Both eluents contain 5 mM ammonium acetate.

### Dual-culture assays

Single colonies of the *Streptomyces* AS1 were inoculated onto ½NA as a 2-cm streak approximately 2 cm from the 9-cm Petri plate edge. After incubation at 25 °C for 48 h, an amount of 5 μl of fungal spore suspension (10^6^ spores/ml) was applied at a distance of 3–4 cm from the *Streptomyces* AS1. *A. fumigatus* assays were incubated at 30 °C and the other assays at 25 °C for 7 days. The inhibition was determined based on the fungal colony area and macroscopic interaction between the dual cultures with each colony given an individual numerical score. These were added up to obtain an overall index of dominance (*I*_D_) as developed by Magan and Lacey (1984). Each interacting species was given an individual score based on the following numerical scores: 1:1—mutual intermingling, 2:2—mutual antagonism on contact, 3:3—mutual antagonism at a distance, 4:0—dominance of one species on contact and 5:0—dominance of one species over the other at a distance. All the experiments were done with three triplicates per treatment and repeated once.

### Statistical analysis

Normal distribution of data was checked by the Shapiro-Wilk *W* test. The general influence of antifungal extract on fungal growth and mycotoxin production were checked using one-way analysis of variance (ANOVA) for normal distribution and the Kruskal-Wallis tests (rank sums) for non-normally distributed data. Student’s *t* test was further applied to compare the means for each treatment for normally distributed data and the Wilcoxon method (non-parametric comparison) for non-normal distribution datasets. A significance level of *p* < 0.05 was used to compare treatment. The JMP Pro (SAS Institute Inc., Cary, NC, USA) was used for these analyses.

## Results

### *Streptomyces* AS1 for control of fungal pathogens using dual-culture assays

**Fig. 1:**
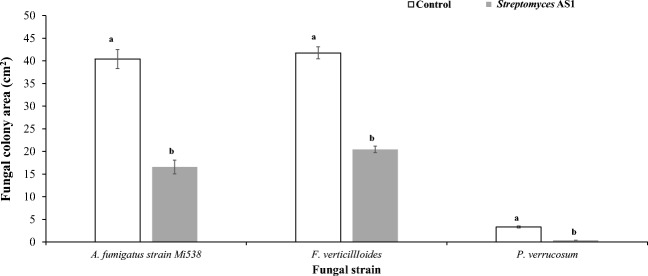
Effect of interaction between *Streptomyces* AS1 and fungal pathogens on the fungal colony area. Each value is a mean of three replicates ± SD. Different letters indicate significant difference (*p* < 0.05) within the treatment

A significant reduction in the colony area (cm^2^) of all the toxigenic fungi occurred in the presence of *Streptomyces* AS1 culture (Fig. 1). The inhibition was best against *P. verrucosum* (90%) followed by *A. fumigatus* strain (Mi538; 59%) and *F. verticillioides* (51%). The interaction score between the *Streptomyces* AS1 and the isolates of all three species was 5:0, indicating dominance at a distance. The total *I*_D_, which was the sum of the individual scores, was thus 15:0 (AS1:mycotoxigenic strain). Indeed, the mycotoxigenic strains of all three species grew away from the *Streptomyces* AS1 strain. There was no increase in the colony area of the pathogens after day 3, especially for *P. verrucosum*.

### Identification of bioactive compounds from the *Streptomyces* AS1 strain

Table 1 shows the major compounds extracted from the ethyl acetate fraction of the *Streptomyces* AS1 strain biomass. There were four major compounds present. These included valinomycin (150 μg/g dry biomass), cyclo(L-Pro-L-Tyr) (22 μg/g), cyclo(L-Pro-L-Val) (10 μg/g) and brevianamide F (3 μg/g). In addition, six very minor compounds common in some food matrices were identified as rugulusovin, tryptophol, chloramphenicol, monactin, dinactin and nonactin (all < 0.7 μg/g; Adetunji et al. 2019).

**Table 1:** Mean concentrations of compounds isolated from ethyl acetate extract of *Streptomyces* AS1 supernatant. The major compounds are in italics

Compounds	Concentration (μg/g dried biomass)
*Valinomycin*	*150*
*Cyclo(L-Pro-L-Tyr)*	*22*
*Cyclo(L-Pro-L-Val)*	*10*
*Brevianamide F*	*3*
Rugulusovin	1
Tryptophol	> 1
Chloramphenicol	> 1
Monactin, dinactin, nonactin	> 1

### Efficacy of *Streptomyces* AS1 metabolites for control of spore germination of *P. verrucosum*, *F. verticillioides* and *A. fumigatus* (strain Mi538)

**Fig. 2:**
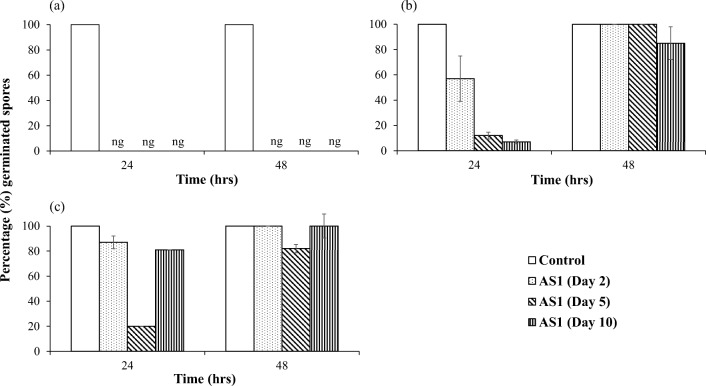
Mean percentage germination of **a***P. verrucosum*, **b***A. fumigatus* (Mi538) and **c***F. verticillioides* spores on half-strength nutrient agar containing *Streptomyces* AS1 metabolites. Data are mean of three replicates ± SD. The data analysis was carried out on the actual data. The percentages were plotted for presentation purposes only. ng no germination

Figure 2 shows the efficacy of the mixture of metabolites secreted by *Streptomyces* AS1 on the germination of conidia of *P. verrucosum*, *A. fumigatus* (Mi538) and *F. verticillioides.* None of the *P. verrucosum* conidia germinated in all the treatment conditions tested. However, 85% of *A. fumigatus* conidia germinated after 48-h incubation. For *F. verticillioides*, there was very little efficacy in controlling microconidial germination.

### Effect of *Streptomyces* AS1 ethyl acetate extract on time to detection of *P. verrucosum*, *A. fumigatus* (Mi538) and *F. verticillioides* using the BioScreen assay method

**Fig. 3:**
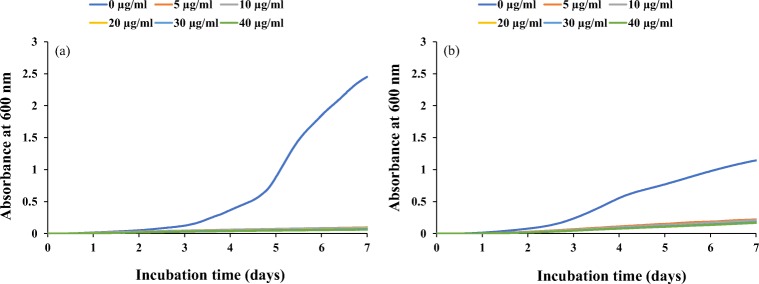
Growth curves of **a***P. verrucosum* and **b***F. verticillioides* at 0.95 *a*_w_ in semi-solid YES media containing different concentrations (0–40 μg/ml) of *Streptomyces* AS1 ethyl acetate extract over 7 days incubation at 25 °C using the BioScreen C. Each curve represents the mean of ten individual replicates

Figure 3 shows an example of the mean growth curve of *P. verrucosum* and *F. verticillioides* obtained after inoculation with different concentrations of *Streptomyces* AS1 ethyl acetate extract at 0.95 *a*_w_ levels over 7 days.

**Fig. 4:**
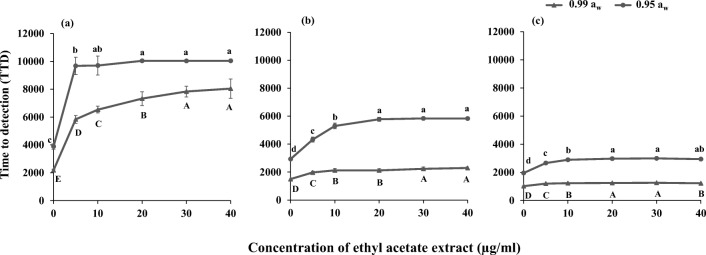
Effect of *Streptomyces* AS1 ethyl acetate extract on the time to detection (TTD; optical density of 0.1 at 600 nm) for **a***P. verrucosum*, **b***A. fumigatus* and **c***F. verticillioides.* Data are mean of ten replicates with bars indicating standard deviation. Different letters indicate significant differences (*p* < 0.05) within treatments

Figure 4 shows the effect of different concentrations of the AS1 ethyl acetate extract at two water activity levels (0.99 and 0.95 *a*_w_) on the time to detection (TTD) at an OD equal to 0.1 for *P. verrucosum*, *A. fumigatus* and *F. verticillioides*. Overall, the TTD of *P. verrucosum* was the highest at both *a*_w_ levels, followed by *A. fumigatus* and *F. verticillioides*. For *P. verrucosum*, AS1 extract at 5 μg/ml resulted in a significant increase (*p* < 0.05) of the TTD at both *a*_w_ levels indicative of effective control of growth. At 0.99 *a*_w_, there was a significant increase in the TTD (*p* < 0.05) until 30 μg/ml after which the effect stabilized. For *A. fumigatus*, with freely available water (0.99 *a*_w_), the TTD was significantly increased until 30 μg/ml concentration. At 0.95 *a*_w_, this was for concentrations up to 20 μg/ml. For *F. verticillioides*, the TTD was significantly increased (*p* < 0.05) with concentrations up to 20 μg/ml of AS1 extract at both *a*_w_ levels.

The minimum inhibitory concentration (MIC) and the IC_50_ concentrations of the AS1 extracts were determined and shown in Table 2. The MIC for *P. verrucosum*, *A. fumigatus* (Mi538) and *F. verticillioides* growth at both *a*_w_ levels was more than the highest concentration tested (> 40 μg/ml). The IC_50_ for *P. verrucosum* suggested that it was the most sensitive with 0.15 μg/ml and 0.005 ng/ml at 0.99 and 0.95 *a*_w_, respectively.

**Table 2:** Minimum inhibitory concentration (MIC) and IC_50_ of the *Streptomyces* AS1 ethyl acetate extract for controlling growth of isolates of *P. verrucosum*, *A. fumigatus* strain Mi538 and *F. verticillioides*

Fungal strain	Water activity (*a*_w_)	MIC (μg/ml)	IC_50_ (μg/ml)
*P. verrucosum*	0.99	> 40	0.15
	0.95	> 40	0.000005
*A. fumigatus* (Mi538)	0.99	> 40	> 40
	0.95	> 40	10.45
*F. verticillioides*	0.99	> 40	> 40
	0.95	> 40	> 40

### Efficacy of *Streptomyces* AS1 ethyl acetate extract on mycotoxin production

The effect of the AS1 extract (Table 3) on mycotoxin production was examined. For *P. verrucosum*, complete control of OTA production was achieved, regardless of *a*_w_ level at the lowest concentration tested (5 μg/ml). For gliotoxin, there was relatively little control by the concentration range tested at both *a*_w_ levels. Indeed, there appeared to be some stimulation at intermediate concentrations. Even at 40 μg/ml, there was no difference between the control and the treatment at both *a*_w_ levels. For *F. verticillioides*, there was a significant reduction (*p* < 0.05) in FB_1_ and FB_2_ at both *a*_w_ levels when compared with the control. AS1 concentrations of 5–40 μg/ml gave similar inhibition of FB_1_ at both *a*_w_ levels and FB_2_ at 0.95 *a*_w_. For FB_2_/0.99 *a*_w_, > 20 μg/ml AS1 was needed to significantly (*p* < 0.05) inhibit production.

**Table 3:** Efficacy of *Streptomyces* AS1 ethyl acetate extract for control of OTA, gliotoxin and fumonisin production by isolates of *P. verrucosum*, *A. fumigatus* strain Mi538 and *F. verticillioides*, respectively. Data are means of three replicates with SE. Different letters (capitals, 0.99 *a*_w_; lowercase letters, 0.95 *a*_w_) indicate significant differences (*p* < 0.05) between treatments by Student’s *t* method for gliotoxin at 0.95 *a*_w_ and the Wilcoxon method (non-parametric comparison) at 0.99 *a*_w_. *ND* none detected

AS1 concentration (μg/ml)	Water activity (*a*_w_)	OTA (μg/g agar)	Gliotoxin (μg/g agar)	Fumonisins (μg/g agar)	
				FB_1_	FB_2_
0	0.99	10.5 ± 4.9	4.1 ± 0.76^A^	3.73 ± 0.07^A^	4.57 ± 0.49^A^
	0.95	13.2 ± 3.1	2.2 ± 0.44^cd^	0.84 ± 0.01^a^	0.96 ± 0.15^a^
5	0.99	ND	6.9 ± 0.30^A^	0.10 ± 0.002^B^	4.20 ± 0.15^A^
	0.95	ND	3.8 ± 0.34^a^	0.06 ± 0.04^b^	0.18 ± 0.04^b^
10	0.99	ND	4.1 ± 0.20^A^	0.09 ± 0.003^B^	4.70 ± 0.66^A^
	0.95	ND	3.4 ± 0.70^ab^	0.03 ± 0.01^b^	0.09 ± 0.02^bc^
20	0.99	ND	7.0 ± 0.03^A^	0.07 ± 0.01^B^	2.26 ± 0.70^B^
	0.95	ND	2.9 ± 0.14^bc^	0.04 ± 0.002^b^	0.07 ± 0.01^bc^
30	0.99	ND	5.0 ± 1.10^A^	0.06 ± 0.003^B^	1.42 ± 0.23^B^
	0.95	ND	1.9 ± 0.06^d^	0.03 ± 0.01^b^	0.07 ± 0.01^c^
40	0.99	ND	4.1 ± 0.15^A^	0.06 ± 0.03^B^	1.32 ± 0.56^B^
	0.95	ND	2.4 ± 0.50^cd^	0.03 ± 0.01^b^	0.08 ± 0.01^bc^

**Fig. 5:**
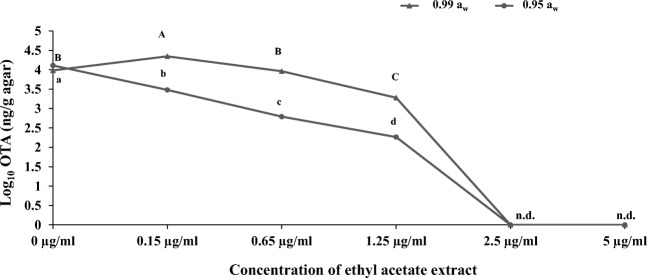
Efficacy of *Streptomyces* AS1 ethyl acetate extract for control of OTA production by *P. verrucosum* at 25 °C. Data are means of triplicates with bars indicating standard errors. Different letters indicate significant differences (*p* < 0.05) within treatments. n.d. none detected

For *P. verrucosum*, because no OTA was detected at 5 μg/ml AS1 extract, further studies with lower concentration of AS1 extract were carried out to identify more accurately the concentrations at which complete inhibition of OTA occurred. Figure 5 shows that there was a significant decrease in OTA production at 0.99 and 0.95 *a*_w_ when the concentration was 1.25 μg/ml and complete inhibition at ≥ 2.5 μg/ml. However, at very low concentrations of 0.15 μg/ml and 0.99 *a*_w_, the OTA production was stimulated when compared with the untreated control.

## Discussion

In the present study, the *Streptomyces* AS1 strain produced compounds which could control the activity of food spoilage mycotoxigenic fungi and an opportunistic human one. In colony-based interactions, the *Streptomyces* AS1 generally inhibited all three species tested with an interaction score of 5:0 indicating dominance at the distance and the production of anti-fungal metabolites. Previous studies with *Streptomyces* species have been associated with the production of primary and secondary metabolites including hydrolytic enzymes and antibiotic-like compounds (Prapagdee et al. 2008; Taechowisan et al. 2005).

The effect of the mixture of *Streptomyces* AS1 metabolites on spore germination of strains of *P. verrucosum*, *F. verticillioides* and *A. fumigatus* was dependent on the time frame of cultivation of the *Streptomyces* AS1 strain. With longer culturing times, more metabolites were produced resulting in better efficacy, especially against *P. verrucosum*. None of the spores germinated under all conditions tested. In contrast, the mixture of compounds was not effective against *A. fumigatus* and *F. verticillioides* although the metabolites did delay germination. Previously, complete inhibition of conidial germination of *A. flavus* spores by metabolites of *Streptomyces* AS1 has been reported (Sultan and Magan 2011).

The efficacy of the mixture of the *Streptomyces* AS1 extract was further examined using a spectrophotometric turbidimetric rapid assay method using the BioScreen C system. The effects of different concentrations of metabolites from the AS1 strain and water activity (*a*_w_) on the TTD for *P. verrucosum*, *F. verticillioides* and *A. fumigatus* and on mycotoxin production were studied. The TTD represents the initial growth phase of the fungi. The shorter the TTD, the less effective the mixture of metabolites were on fungal growth. Overall, the AS1 mixed extract was more effective in controlling growth of *P. verrucosum*, even at low concentrations. This paralleled the findings on effects on conidial spore germination inhibition of the strain of this species. More importantly, the mixed AS1 extract suppressed production of OTA by *P. verrucosum* and also reduced the production of FB_1_ and FB_2_ by *F. verticillioides* at both *a*_w_ levels examined. Indeed, OTA was not detected when 5 μg/ml of the the AS1 extract was used. Thus, more detailed efficacy testing was done to determine the lowest concentrations which could be used to inhibit OTA production. Complete inhibition of OTA production was achieved at very low concentrations (2.5 μg/ml). Thus, the predominant metabolites produced by the *Streptomyces* AS1 were fungistatic against spore germination and growth of *P. verrucosum*, and the IC_50_ concentrations were < 0.20 μg/ml and resulted in complete inhibition of OTA mycotoxin production at both *a*_w_ levels tested. However, this did not occur with the *A. fumigatus*, responsible for aspergillosis of the lungs, where there was no control of gliotoxin production.

Previous studies with other *Streptomyces* strains have suggested no effect on *P. verrucosum* growth, only some inhibition of *A. fumigatus*, but good efficacy against *F. verticillioides* (Nguyen et al. 2018; Paškevičius et al. 2014). However, previous studies only focused on control of mycelial growth and not on mycotoxin production. A previous study by Medina et al. (2007) found compounds from another *Streptomyces* strain were effective in controlling *Aspergillus carbonarius* growth and OTA production.

A total of 10 compounds were found to be present in the AS1 ethyl acetate extract. The major compound present was valinomycin (150 μg/g). Three others present which may have contributed to the anti-fungal activity were cyclo(L-Pro-L-Tyr), cyclo(L-Pro-L-Val) and brevianamide F. Comparisons were made between efficacy of the mixed AS1 extract and valinomycin and cyclo(L-Pro-L-Tyr) alone and as a mixture, for which standards are available. These were found to have no effect on growth of the target species when compared with the mixed AS1 metabolites (MohD Danial 2019). This suggests that the combined mixture has much better efficacy than the major individual compound or a mixture of the two main compounds alone.

The production of these compounds by other *Streptomyces* species and bacteria such as *Bacillus* sp. N strain, *Pseudomonas aurantiaca* and *Cellulosimicrobium cellulans* has been reported previously (Buedenbender et al. 2018; Gwee Kyo et al. 2011; Kumar et al. 2013; Li et al. 2006; Park and Shim 2014; Park et al. 2008, Wattana-Amorn et al. 2016). The other compounds found were mainly cyclic ionophores which were present in very low amounts and thus probably did not directly contribute to the anti-fungal activity. Previous studies have shown variable results in terms of efficacy depending on the target fungal genera or species including *Aspergillus*, *Fusarium*, *Penicillium*, *Rhizoctonia* and *Candida* species (Gwee Kyo et al. 2011; Kumar et al. 2013; Park and Shim 2014; Park et al. 2008). Although brevianamide F was a relatively minor component, its potential role in anti-fungal activity has not been previously described.

In conclusion, the mixture of metabolites produced by the *Streptomyces* AS1 species was more effective in suppressing spore germination and mycelial growth of *P. verrucosum* than of *F. verticillioides* or *A. fumigatus.* A very low concentration of AS1 extract was able to inhibit mycelial growth of *P. verrucosum* by 50% although > 40 μg/ml of the mixture of compounds was needed for complete inhibition. The mixture of metabolites produced by the *Streptomyces* AS1 successfully inhibited OTA production by *P. verrucosum* completely at concentrations of 2.5 μg/ml. The potential for using the mixture of these metabolites now needs to be examined in situ in stored temperate cereals to examine the efficacy for control of colonization by *P. verrucosum* and OTA contamination during short- and medium-term storage.
